# Pre-pandemic trends and Black:White inequities in life expectancy across the 30 most populous U.S. cities: a population-based study

**DOI:** 10.1186/s12889-023-17214-1

**Published:** 2023-11-22

**Authors:** Abigail Silva, Nazia S. Saiyed, Emma Canty, Maureen R. Benjamins

**Affiliations:** 1 Parkinson School of Health Sciences and Public Health, Loyola University Chicago, Chicago, IL USA; 2 Sinai Urban Health Institute, Chicago, IL USA

**Keywords:** Life expectancy, Mortality, Race, Racism, Structural racism, Inequities, Disparities, Health equity, Place, Urban

## Abstract

**Background:**

Racial inequities in life expectancy, driven by structural racism, have been documented at the state and county levels; however, less information is available at the city level where local policy change generally happens. Furthermore, an assessment of life expectancy during the decade preceding COVID-19 provides a point of comparison for life expectancy estimates and trends post COVID-19 as cities recover.

**Methods:**

Using National Vital Statistics System mortality data and American Community Survey population estimates, we calculated the average annual city-level life expectancies for the non-Hispanic Black (Black), non-Hispanic White (White), and total populations. We then calculated the absolute difference between the Black and White life expectancies for each of the 30 cities and the U.S. We analyzed trends over four time periods (2008-2010, 2011-2013, 2014-2016, and 2017-2019).

**Results:**

In 2017-2019, life expectancies ranged from 72.75 years in Detroit to 83.15 years in San Francisco (compared to 78.29 years for the U.S.). Black life expectancy ranged from 69.94 years in Houston to 79.04 years in New York, while White life expectancy ranged from 75.18 years in Jacksonville to 86.42 years in Washington, DC. Between 2008-2010 and 2017-2019, 17 of the biggest cities experienced a statistically significant improvement in life expectancy, while 9 cities experienced a significant decrease. Black life expectancy increased significantly in 14 cities and the U.S. but decreased significantly in 4 cities. White life expectancy increased significantly in 17 cities and the U.S. but decreased in 8 cities. In 2017-2019, the U.S. and all but one of the big cities had a significantly longer life expectancy for the White population compared to the Black population. There was more than a 13-year difference between Black and White life expectancies in Washington, DC (compared to 4.18 years at the national level). From 2008-2010 to 2017-2019, the racial gap decreased significantly for the U.S. and eight cities, while it increased in seven cities.

**Conclusion:**

Urban stakeholders and equity advocates need data on mortality inequities that are aligned with city jurisdictions to help guide the allocation of resources and implementation of interventions.

**Supplementary Information:**

The online version contains supplementary material available at 10.1186/s12889-023-17214-1.

## Introduction

Life expectancy at birth, perhaps the best single indicator of overall health in a population, summarizes mortality from all diseases and injuries, and across all ages [1, 2]. Racial inequities in this important health metric have consistently been observed at the national level since at least 1850, when a White person could expect to live to age 40, on average, while a Black person could only expect to live to age 23 [3]. Although this gap varied throughout the 1900’s, it generally narrowed through the end of the century and early 2000’s [2, 4]. In the first two decades of the twenty-first century, the Black-White difference in life expectancy shrank by more than two years (from 5.9 to 3.6 years) [5]. However, the racial gap increased by almost two years (to 5.50 years in 2021) due to the COVID-19 pandemic, reflecting continued systemic racism that disproportionately affects communities and individuals of color [6–9].

Racial inequities in life expectancy have also been documented at the state and county levels; [10, 11] however, less information is available at sub-county levels, including cities. Public health officials and researchers increasingly seek local data to inform place-based initiatives [12–14]. This is especially important because data for bigger geographic areas (e.g. county) hides local variation, making it difficult to detect inequities [15]. Even city-level data hides variation across neighborhoods; [16] however, local policy change generally happens at the city (not neighborhood or census tract) level, making it an optimal level for analysis. Moreover, four in five people in the U.S. currently live in urban areas and this number continues to grow [17].

In the past several years, there has been increasing access to city-level health data via open access data platforms [18, 19]. However, life expectancy estimates at the city level are either based upon small area estimates and do not include race-specific estimates, [18] or the race-specific sources do not estimate *non-Hispanic* Black life expectancy [19]. In addition, they include very limited (or no) historical data and lack an an evaluation of trends. It is critical to add to this work by specifically examining inequities between non-Hispanic Black (NHB) and non-Hispanic White (NHW) populations and assessing how these inequities have changed during the past decade leading up to the COVID-19 pandemic (prior to dramatic shifts in life expectancy). The explicit measurement of racial inequities and city-level comparisons of such inequities provide a more in-depth assessment of our current urban equity landscape [20] In addition, trend analyses can help identify cities that have been making significant progress in improving population health and/or ameliorating inequities [21, 22]. These cities can serve as potential models to others that are striving to be more equitable. Furthermore, an assessment of life expectancy during the decade preceding COVID-19 provides a point of comparison for life expectancy estimates and trends post COVID-19 as cities recover.

This analysis provides data on total and race-specific life expectancy, and inequities found within, for the 30 most populous U.S. cities. We assess trends in inequities across four time periods between 2008 and 2019. These new data provide critical information for urban health leaders and equity advocates to help identify areas and groups of greatest need, motivate partners, and begin exploring ways to address equity challenges through population-level structural interventions [22–24].

## Methods

This study followed the Strengthening the Reporting of Observational Studies in Epidemiology reporting guideline for observational studies.

### Study population

The 30 most populous cities were identified according to the 2019 U.S. Census Bureau data. Our analysis included three cases in which city and county governments representing slightly different geographies have merged to form a consolidated city with one governing body (i.e. Louisville and Jefferson County, KY; Nashville and Davidson County, TN; and Indianapolis and Marion County, IN). In those three cases, mortality data was only available at the county level. Therefore, the results presented for these cases represent county life expectancies. Due to uncertainty about the geographic area included in Las Vegas death records, we excluded Las Vegas from the analysis and included Milwaukee, WI, the 31^st^ largest city, in its place. Collectively, these cities make up 12.1% of the US population. Select demographic data for each city are listed in Supplemental Table 1.

### Data sources

#### Mortality data

Mortality data comes from the Multiple Cause of Death data files from the National Vital Statistics System [25] For the years 2008–2010 (T1), 2011–2013 (T2), 2014–2016 (T3), and 2017–2019 (T4), we extracted race- and ethnicity-specific deaths by age group (0–4, 5–9, 10–14, 15–19, 20–24, 25–29, 30–34, 35–44, 45–54, 55–64, 65–74, 75–84, and 85 years and above) and the recorded city of residence. City of residence (as opposed to city of occurrence) was used to classify the geography for deaths to ensure our numerators and denominators reflected the same population.

#### Population data

For the U.S. overall and each individual city, total race- and age-specific population-based denominators were obtained from the U.S. Census Bureau. Denominator data for the total and non-Hispanic White populations came from the American Community Survey (ACS) 5-year estimates from the midpoint year of each of the time periods included in the study [26]. This is consistent with previous work and methodology used by others in this field [27–29]. One exception was made for Detroit, where ACS estimates for 2009 were inconsistent with estimates for other years. Therefore, for T1 we used the average of the 3-year ACS estimate for 2008 and 5-year ACS estimate for 2010 for the city of Detroit. The process for obtaining non-Hispanic Black denominator data was different as the only source of population data for the *non-Hispanic* Black population is the Decennial Census. The non-Hispanic Black population was estimated for the U.S. overall and each individual city using the formula:

$$NHB\;Pop\;Estimate = \sum_{n=1}^{13}\left\{\frac{\left[{NHB\;Pop}_{US\;Census}\right]}{\left[{Black\;Pop}_{US\;Census}\right]}\times \left[{Black\;Pop}_{ACS}\right]\right\}$$

Where *n* is age group, *NHB Pop* is Non-Hispanic Black Population, *Black Pop* is Total (Hispanic + non-Hispanic) Black Population, *US Census* refers to the 2010 Decennial and ACS refers to the American Community Survey 5-year estimates for the midpoint year of each study period.

The total overall U.S. and individual city outcomes include all race/ethnic groups (not just Black and White). To calculate life expectancy, we used all 13 age groups available in the ACS.

### Statistical analyses

#### Life expectancy

Life expectancy measures the average number of years from birth a person can expect to live according to the current mortality experience (age-specific death rates) of the population. Life expectancy was calculated for the total population, the NHB (Black) population, and the NHW (White) population. The life expectancy for each city and the U.S. overall were calculated using a modified version of the life expectancy calculation developed by Chiang [30]. Chiang’s original life expectancy formula was calculated using 18 distinct age groups, whereas our life expectancy formula was restricted to the 13 age groups available in the ACS. To assess the accuracy of this method, a test was conducted wherein life expectancy was calculated for the same populations using 13 and 18 age groups, with data from the 2010 U.S. Census. Both methods produced similar results, therefore, the ACS data was used so to have population denominators that reflected the same time periods as the deaths included in this analysis.

Z scores were calculated to assess the significance of changes in life expectancy over time for the total populations, as well as Black and White populations. To assess inequities in life expectancy, we calculated the absolute difference in Black and White life expectancy and its 95% confidence interval for each of the 30 cities and the U.S. overall in each time period. To identify a statistically significant increase or decrease in life expectancy inequities, the *t-*test was applied.

This study was reviewed by the Mount Sinai Hospital (Chicago, IL) institutional review board (IRB) and deemed to be exempt, in accordance with 45 CFR §46. Mount Sinai Hospital IRB waived written informed consent due to the retrospective and deidentified nature of the data.

## Results

A total of 31,686,825 death records were assessed for eligibility, from which 62,671 records of non-U.S. residents and 1,799 records where age was missing were excluded. There were 31,622,355 death records from 2008–2019 included in the analysis.

### Overall life expectancy

The life expectancy for a baby born in the U.S. between 2017–2019 (T4) was 78.29 years, as seen in Table 1. Life expectancy at the city level ranged from a low of 72.75 years in Detroit to a high of 83.15 years in San Francisco. Of the 30 cities, 13 had life expectancies higher than the U.S., while 17 had lower life expectancies.

**Table 1:**
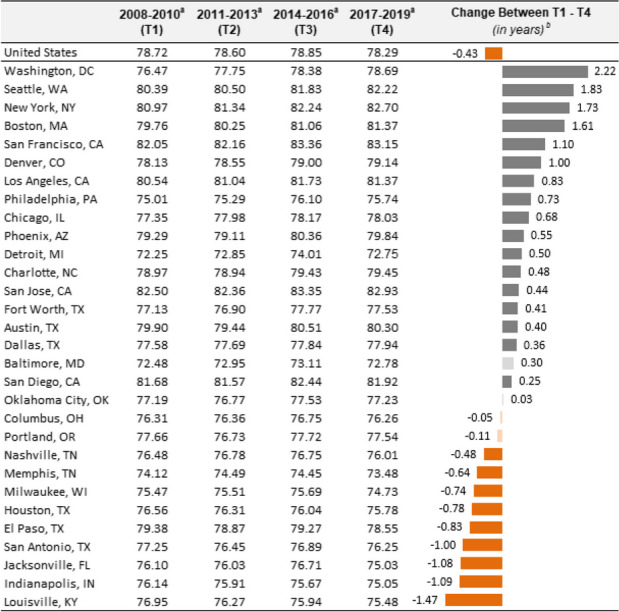
Trends in life expectancy for the U.S and the 30 most populous cities

^a^Life expectancy at birth, in years

^b^Darker bars represent statistical significance (*p* < .05)

The U.S. experienced a small but significant decrease in life expectancy (from 0.43 years; *p* < 0.05) between T1 and T4 (see Supplemental Table 2 for all time points). Also, nine cities experienced a statistically significant decrease in life expectancy, ranging from a loss of 0.48 years in Nashville to 1.47 years in Louisville. Conversely, 17 experienced a statistically significant improvement in life expectancy. This increase ranged from 0.25 years (San Diego) to 2.22 years (Washington DC). Life expectancy in the remaining four cities did not change.

### Race-specific life expectancy

During the most recent time period (T4), the Black life expectancy for the U.S. was 74.25 years (Table 2). Across the cities, life expectancy for Black individuals ranged from 69.94 years in Houston to 79.04 years in New York. The White life expectancy for the U.S. was 78.44 years. The range across cities (11.24 years) was slightly larger in magnitude to that seen for Black life expectancy (9.10), going from 75.18 years (Jacksonville) to 86.42 years (Washington, DC).

**Table 2:**
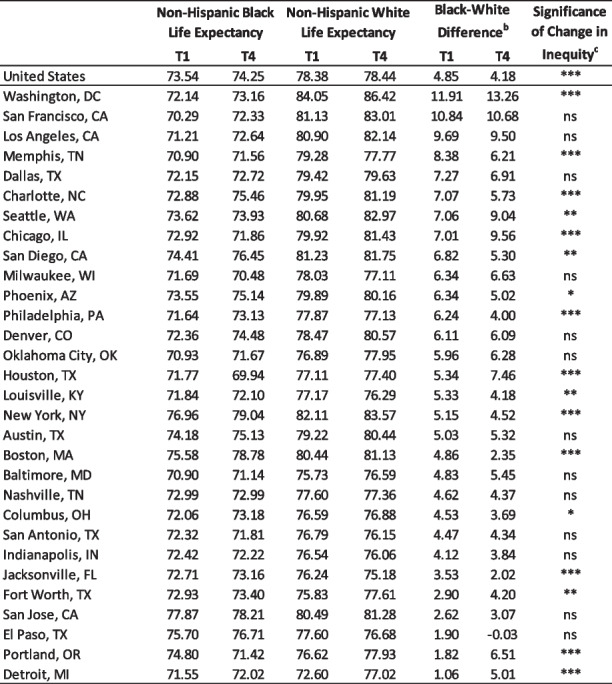
Black and white life expectancy in 2008-2010 (T1) and 2017-2019 (T4)^a^

^a^Life expectancy at birth, in years

^b^Black-white difference was calculated by subtracting Black Life Expectancy from White Life Expectancy

^c*^p < 0.05, ^**^p < 0.01, ^***^p < 0.0001. *ns* Not statistically significant

Black life expectancy increased significantly from T1 to T4 in the U.S. (from 73.54 to 74.25 years) and in 14 of the 30 largest cities (see Supplemental Table 2 for all time points). This increase in Black life expectancy ranged from 0.57 years (Dallas) to 3.20 years (Boston). However, life expectancy for the Black population significantly decreased in four cities (Portland, Houston, Milwaukee, and Chicago). The lowest decrease occurred in Chicago (1.05 years) and the highest in Portland (3.38 years). There was no statistically significant change in Black life expectancy in the 12 remaining cities.

White life expectancy in the U.S. showed a small, but statistically significant, increase from 78.38 to 78.44 years between T1 and T4 (see Supplemental Table 2 for all time points). Among the big cities, it increased significantly in 17 cities (ranging from an increase of 0.51 years in San Diego to 4.42 years in Detroit) and decreased in 8 cities (ranging from a decrease of 0.48 years in Indianapolis to 1.51 years in Memphis). There was no statistically significant change in White life expectancy in the remaining five cities (Nashville, Dallas, Phoenix, Houston, and Columbus).

### Racial inequities in life expectancy

At the national level, the absolute difference between the Black and White life expectancies was 4.18 years in T4. All but one city (El Paso) had a longer life expectancy for the White population compared to the Black, with most (*n* = 27) showing a difference of three or more years between the race-specific rates. In 22 of the 30 cities, the racial difference was wider than the one found at the national level. Most strikingly, there was more than a 13-year difference (13.26 years, 95% CI: 12.80, 13.72) between Black and White life expectancies in Washington, DC.

**Fig. 1:**
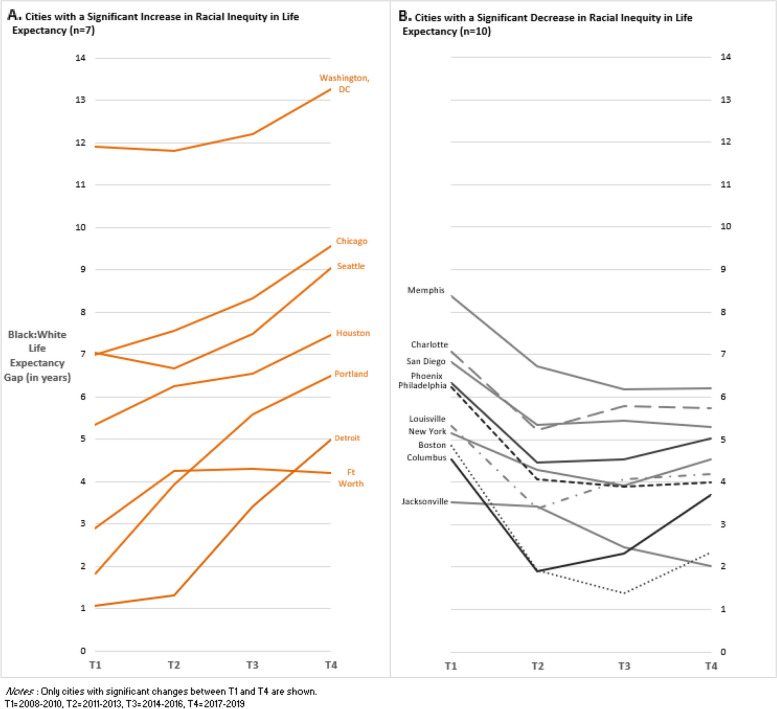
Size of black: white life expectancy gap across four yime periods

In the U.S., the inequity statistically significantly decreased by 0.67 years for the country from T1 to T4, with inequity declining more sharply between T1 and T2, and then remaining stable from T2 to T4. In Fig. 1, we plotted the change in Black:White life expectancy inequity over time for the cities in which there was a significant change from T1 to T4. There were significant increases in the Black:White difference in seven cities. Of note, Houston, Chicago, Detroit, and Portland all saw increases in the Black:White gap of more than two years between T1 and T4. The cities where inequities increased generally experienced increases over the four time periods, except for Fort Worth, where inequity increased sharply from T1 to T2 then remained flat for the remainder of the study period. In contrast, the inequity decreased significantly between T1 and T4 in ten cities, with decreases of more than two years in Boston, Philadelphia, and Memphis. Among these cities, the data showed a trend of sharp decreases between T1 and T2, followed by modest increases from T2 through T4. Jacksonville was an exception to this trend; it was the only city where inequities decreased at each point during the study period. The racial inequity remained stable in 13 cities.

### Comparing cities by outcome and equity

**Fig. 2:**
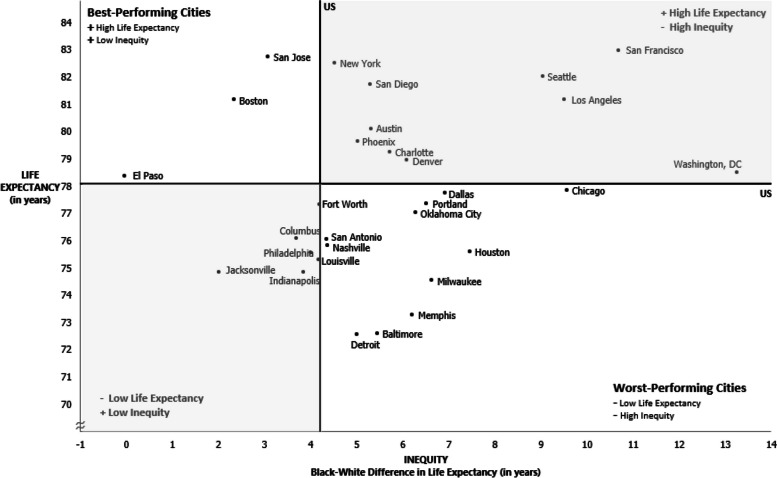
Life expectancy and racial inequity in life expectancy (2017–2019)

In Fig. 2, we plotted the 30 cities based on both overall life expectancy (y-axis) and the racial gap in life expectancy at T4 (x-axis). The U.S. life expectancy and Black:White difference in life expectancy were used to separate outcomes into quadrants. The upper left quadrant represents the “best-performing cities” that had life expectancies above the U.S. life expectancy and Black:White differences below the national level of inequity. El Paso, Boston, and San Jose are the only cities in this category. The lower right quadrant represents the “worst-performing” cities that had life expectancies lower than the U.S. and levels of racial inequity greater than the U.S. gap. There are 11 cities that performed poorly in both outcomes. Chicago stands out for high inequity, while Detroit, Baltimore, and Memphis stand out for having poor overall life expectancy within this group.

The remaining cities performed well for *either* life expectancy or equity. Most notably, San Francisco was the top city for longest life expectancy but the second worst city for racial inequity. Conversely, Jacksonville was one of the top cities for racial equity in life expectancy but was well below the national life expectancy.

## Discussion

Urban stakeholders and public health professionals have increasingly called for more granular data to identify local health issues, compare outcomes, and assess progress [13, 18, 31]. The present study provides detailed city-level data on racial inequities in life expectancy across four time points, confirming wide variation among the biggest U.S. cities. Across 29 of the biggest cities and all time points (El Paso being the exception), Black life expectancy was consistently shorter than White life expectancy. In the most recent time period (2017–2019), prior to the COVID-19 pandemic, 18 of the 29 cities had a Black-White gap in life expectancy of at least 5 years and the remainder had at least a 2-year gap. The size of the inequities, despite decades of programs and policies designed to eliminate them, is troubling. Even more worrisome is the trend of increasing inequities in seven cities, with another 15 cities showing a consistent racial gap over time. Overall, the data suggest that the U.S. needs a more tailored approach to achieve health equity in our urban areas.

The current data adds to existing sources of city-level data health information. For example, the City Health Dashboard provides life expectancy estimates for the 900 largest U.S. cities, but they do not currently include race-specific data, their numbers are estimated using weighted averages of small area estimations of life expectancy, and they only include a single six-year estimate [29]. A second notable source of data, the Big Cities Health Inventory, provides extensive health data for the 35 largest U.S. cities. However, the mortality data provided there for the Black population includes individuals with either Hispanic or non-Hispanic ethnicity [19]. Race and ethnicity are two separate social constructs and research shows that Black Hispanic and Black non-Hispanic populations have different health experiences [32–34]. Within the peer-reviewed literature, a relatively recent paper provided an extensive analysis of trends in (all-cause and cause-specific) mortality and life expectancy at the city level. However, the authors did not include explicit measures of racial inequities and used county-level data for race-specific life expectancy estimates [19].

City departments of public health guide large budgets, set the agenda for local health initiatives, and often lead innovative policy development [35, 36]. However, conducting this type of local-level analysis is time-intensive and requires methodological expertise, which may be beyond the capacity of local public health departments due to funding constraints [37–39]. Thus, cities are often compelled to make decisions based on data not specific to their actual population. Furthermore, a growing number of local health departments are specifically focused on increasing racial health equity in their jurisdiction, as indicated by the inclusion of racial health equity within strategic health plans, the establishment of equity-related positions or divisions, the provision of health equity data, and declarations of racism as a public health crisis [40, 41]. An evaluation of these strategic plans and activities must include a regular assessment of racial inequities in health outcome measures, as was done in this study. Cities that increase their focus (and presumably budget) on equity-related outcomes should carefully evaluate trends in their local data to ascertain if the additional resources translate into decreased racial gaps over time. If not, the data could be used to strengthen appeals for increased support, as well as to prompt a critical examination of the strategies used.

More broadly, this type of data offers the opportunity to comparatively assess local policies, programs, and resources that may be supportive of more equitable health outcomes. Our study identified cities that had below average racial inequities, as well as those making strides towards greater equity. In particular, both Jacksonville and Boston had a smaller Black:White gap than the country as a whole and a significant reduction in the size of the gap between T1 and T4. In a recent study covering the local health departments that serve the 30 biggest US cities, these same two cities were recognized for having both a focus on racial health inequities within their strategic plan and having specific goals related to racial health equity [42]. While many other factors, including structural racism (e.g., income inequality), influence population-level outcomes like inequities in life expectancy, the impact of these types of policy-related indicators warrants more investigation [27].

Moving forward, analyses like the current study may also spur analyses at an even more local level to pinpoint the neighborhoods or population subgroups that would benefit the most from targeted interventions or resource allocations [43, 44]. Another potentially useful approach is for cities to decompose life expectancy inequities to determine which causes of death contribute the most to the racial gaps, which could help individuals cities more effectively target potential solutions [45, 46]. For cities with health inequities, evidence-based or -informed interventions and policies that address social determinants of health and are intentionally directed at reducing health inequities are critical for closing the racial gap in health [47–50] There are several model city-level initiatives that offer insight into how local data and multi-sector approach may be used to tackle and assess initiatives aimed at addressing racial inequities in care and outcome [51–53]

This work has several limitations. To begin, the race and ethnicity data provided on death certificates may be inaccurate as it may come from interviews with the decedent’s family or on the reporter’s observations. However, research suggests race reporting for the categories used here (White and Black races and Hispanic ethnicity) is highly accurate [54, 55]. We also recognize the complexity of quantifying inequities and acknowledge that we only include one measure of inequities [56]. Additionally, we limited our analyses to inequities between the Black and White populations. We made this choice because, in the U.S., Black and White populations are frequently used in public health as representative of the extremes of privilege and marginalization [27]. Future work should examine inequities affecting other racial and ethnic groups, as data permit. Finally, the current analyses do not include an examination of ecological factors and, thus, cannot account for demographic or socioeconomic changes that may have occurred within the cities during the study period.

## Conclusions

It is critical to measure racial inequities in life expectancy and assess how they are changing over time in order to gauge city-level progress in achieving health equity, a primary goal within the Healthy People initiative [57] and a specific focus of many health departments covering our largest urban areas [41]. Moreover, directly comparing (or even ranking) racial inequities across geographic areas can increase awareness and advocacy for equity; guide resource allocation; and highlight cities that might be implementing effective population health strategies [58–63]. The pandemic’s short-, mid-, and long-term impacts on life expectancy in urban cities remains to be seen. A re-assessment of life expectancy trends during and after the COVID-19 pandemic is warranted given that populations in urban centers were amongst the hardest hit [64]. Additionally, local-level variability and inequities in vaccination uptake are likely to forestall improvement and equity in life expectancy in some areas [65]. Structural racism and social conditions facilitate inequities in life expectancy (and many other health outcomes). This is reflected in the persistent racial gaps seen in almost all the cities included in the present analysis. The magnitude and multifaceted nature of these inequities demand broad and immediate economic, social, and political changes that allow everyone equal opportunities to leading healthy lives [6, 66, 67].

### Supplementary Information

**Additional file 1: Table 1.  **Selected City-Level Sociodemographic Characteristics for the U.S. and the 30 Most Populous Cities.

**Additional file 2: Table 2. **Life Expectancy and 95% Confidence Intervals for the Total Population (Males and Females) by Racial Group at Four Time Points.

## Data Availability

The datasets analyzed during the current study are restricted-use vital statistics data obtained from the National Vital Statistics System (NCHS) via a data use agreement. Data are available from the authors upon reasonable request and with permission from NCHS.
